# Diagnosis and treatment for central Mucoepidermoid Carcinoma in the mandible: Report of a clinical case in a young patient

**DOI:** 10.4317/jced.55919

**Published:** 2019-12-01

**Authors:** Yuritza Hernández-Arenas, Efraín Del Cristo Álvarez-Martinez, Carlos-Martin Ardila

**Affiliations:** 1 Posgraduate student in Cirugía Oral and Maxilofacial, Facultad de Odontología, Universidad de Antioquia. Medellín, Colombia; 2 Titular Professor Facultad de Odontología, Universidad de Antioquia. Medellín, Colombia; 3 Ph.D. in Epidemiology; Biomedical Stomatology Research Group, Universidad de Antioquia, Medellín, Colombia

## Abstract

A clinical case of a 13 year old male patient with a malignant tumor in the mandibular body area with two years of evolution and associated pain is reported. It was initially diagnosed as low grade central mucoepidermoid carcinoma through Hematoxylin and Eosin (H-E) staining in multiple biopsies. Considering the clinical and radiographic characteristics of the lesion, it was necessary to confirm the diagnosis through Periodic acid-Schiff (PAS). The treatment involved hemimandibulectomy, neck emptying, and complementary radiotherapy. This article aims to present a rare occurrence of this type of intraosseous malignant tumor of glandular origin in a young patient.

** Key words:**Mucoepidermoid carcinoma, periodic acid Schiff reaction, pathology, surgery, radiotherapy.

## Introduction

Mucoepidermoid carcinoma (MEC) is the malignant salivary gland neoplasm mainly presented in the minor salivary gland. One of the occasional variations of MEC is intraosseous alternative located in the jaws identified as central mucoepidermoid carcinoma (1). It was first described in the mandible of a 66 year old woman (2). Later, two cases discussing the criteria of their origin, histological composition, and possible explanations for tumor pathogenesis were published (3). Subsequently, in 1991 (4), the term mucoepidermoid carcinoma was suggested. In 2008, MEC was included in the category of intraosseous injuries, and its differential diagnosis was established under clinical, histological, and radiological features (5).

Most of central MEC appear in the mandible and seldom observed in the maxilla. Primary intraosseous (central) MECs are rare in young people (1). A recent review presented 147 cases, and only five of them involved patients less than 15 years (6). This article aims to show how this pathology can appear in young people and a low-prevalence area, making the clinical and histopathological diagnosis difficult.

## Case Report

A 13 year old male patient consulted with a clinical picture of 2 years of evolution, related to enlargement in the left hemimandibula, mild pain, edema, and induration; signs and symptoms associated with the delayed eruption of the posterior ipsilateral molar.

**Figure 1:**
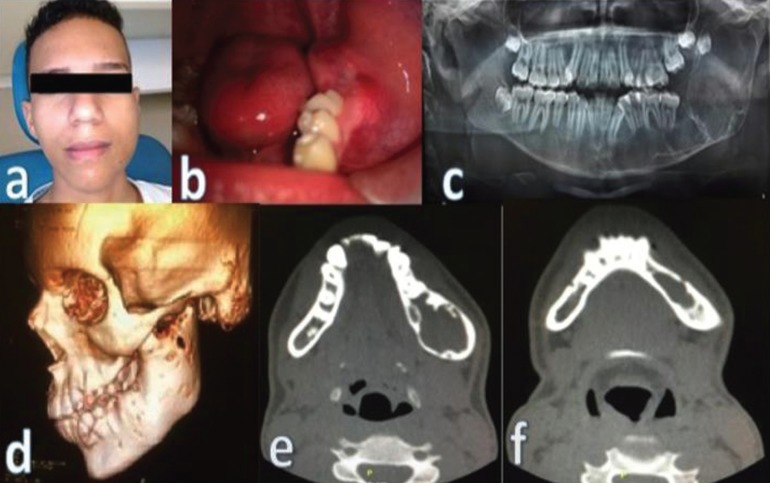
a) Facial asymmetry b) Expansion of bone plates c) A large radiolucent area divided by thick radiopaque lines. d. e. f. ) Bone cortical expansion.

During the extraoral physical examination, facial asymmetry was observed by volume increase in the left mandibular body and angle; the patient complained of paresthesia from the beginning. Presence of bilateral, mobile, submandibular adenopathies with less than 1 cm was also detected (Fig. 1a). Intraorally, cortical expansion in the left retromolar area with a slight pain on palpation in the lingual plate, a stony consistency in an area covered by healthy mucosa was observed (Fig. 1b).

In the initial panoramic radiograph, a large radiolucent area divided by thick radiopaque lines that extend from the left mandibular parasinphysis to the condylar neck and coronoid process was detected. Tooth 38 was displaced until the coronoid area (Fig. 1c). Mandibular reconstruction image (Fig. 1d) and coronal CT cuts (Fig. 1e,f) demonstrate expansion and cortical thinning.

Ameloblastoma, keratocyst, and dentigerous cyst were established as presumptive diagnoses according to clinical and radiographic features. Computerized Axial Tomography (CT) scans of the face with 3D reconstruction, fine needle aspiration biopsy, incisional biopsy, decompression, and histopathological studies were prescribed.

The CT reports a bone lesion with epicenter in the left hemimandibula, compromising the condylar neck, ramus, angle, and hemibody; it was associated with cortical thinning (Fig. 1e,f).

In the first intervention, fine needle aspiration of 3 cc of citrine fluid was performed, which continued with a hematic fluid output. Additionally, an incision was implemented at the level of tooth 37. After the section of periosteum, a thinning of the vestibular plate was observed; bone samples were taken for histopathological study. Besides, a drain was left in the surgical area for a week.

**Figure 2:**
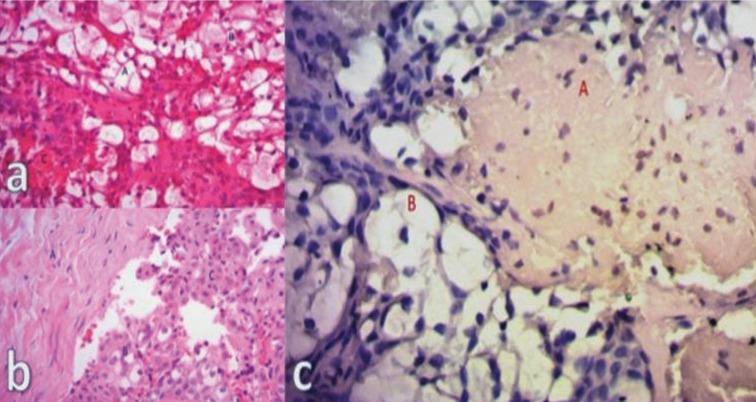
a) Staining with hematoxylin and eosin (H-E). Cells with ample and clear cytoplasm (A). Eosinophilic cytoplasm (B). Hemorrhagic area (C). b). Staining with hematoxylin and eosin (H-E). Dense connective tissue (A). Cells with ample and clear cytoplasm (B). Eosinophilic cytoplasm (C). c). Periodic acid-Schiff (PAS). Positive for mucin (A). Cells with ample and clear cytoplasm (B). 40X.

The histopathological study reported proliferation of neoplastic epithelial cells (cells with large clear cytoplasm, eosinophilic cytoplasm, and squamous appearance) (Fig. 2a-c). There was no differentiation to odontogenic tissue, establishing the diagnosis of low grade central mucoepidermoid carcinoma.

Considering this information, the pathologist suggested performing a Periodic acid-Shiff (PAS) to determine mucin production; this is a reliable confirmatory test, and it is more affordable than immunohistochemistry. The PAS results were positive (Fig. 2c).

**Figure 3:**
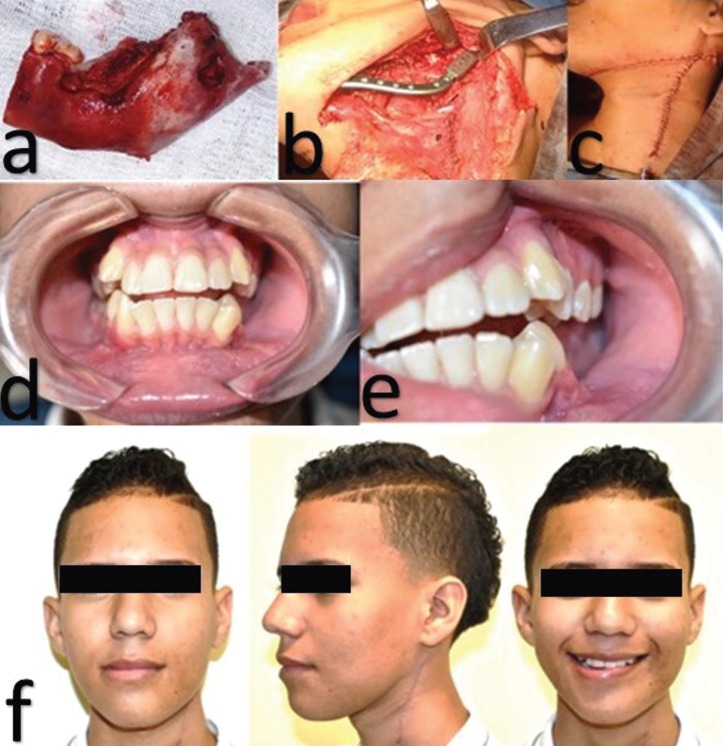
a). Jaw block removed. b). Mandibular reconstruction plate. c). Immediate post-operative. d. e ). Intraoral one-year postoperative. f). Extraoral one-year postoperative.

A low‑grade central mucoepidermoid carcinoma diagnosis was confirmed; therefore, surgical management of the patient was decided (Fig. 3a-c). The patient underwent clinical and radiographic monitoring for one year, without signs of new injuries (Fig. 3d-f).

Written consent of the parents patient according to ethical principles, was signed.

## Discussion

Intraosseous salivary gland tumors are infrequent where MEC has the higher occurrence, which includes 60% of the central salivary gland tumors; following in incidence is the adenoid cystic carcinoma (18%), then acinic cell carcinoma (4%), among others (6-11).

The pathogenesis of central MEC remains unclear; however, several theories have been reported: neoplastic conversion, embryonic remainders glands, and entrapment of the retromolar mucous glands (8-12).

In this case, a 13 year old boy was diagnosed with a low grade central MEC. However, people between the fifth and sixth decade of life, a high prevalence of this tumor has been reported. Thus, this occurrence is rare in a teenager (6).

Six criteria must be considered to establish the diagnosis of central MEC (13,14). Although cortical integrity is one of them, the central origin within the bone may be conditional in the absence of a prominent peripheral soft tissue despite cortical perforation (15).

Regarding the clinical features of central MEC, unlike other malignant tumors in jaws, it appears mainly as a cyst or as a benign tumor. Paresthesia of the inferior alveolar nerve and dissemination to lymph nodes has also been described (14,15).

Radiographically, it seems as a unilocular or multilocular mass with delimited and well-corticated margins. The cases reporting cortical disruption are rare (15).

Histologically, MEC is a malignant epithelial tumor formed by a variable proportion of mucosal, epidermoid, intermediate, columnar, and clear cells, which frequently present a cystic component (6).

The mainstay of the treatment for central MEC is surgery with safety margins. Radical treatment, such as segmental resection with and without adjuvant therapy reports a recurrence rate of 4 % (6).

MEC is a rare condition in teenagers since MEC represents the most common malignancy of salivary glands in pediatric patients; however, an early diagnosis is very important to carry out the treatment as soon as possible.
